# OmicNavigator: open-source software for the exploration, visualization, and archival of omic studies

**DOI:** 10.1186/s12859-024-05743-4

**Published:** 2024-04-24

**Authors:** Terrence R. Ernst, John D. Blischak, Paul Nordlund, Joe Dalen, Justin Moore, Akshay Bhamidipati, Pankaj Dwivedi, Joe LoGrasso, Marco Rocha Curado, Brett Warren Engelmann

**Affiliations:** 1 AbbVie Inc., 1 North Waukegan Rd, North Chicago, IL 60064 USA; 2 AbbVie Deutschland GmbH & Co. KG, Ludwigshafen, Germany; 3 Current Address: Program in Quantitative and Computational Biosciences, Baylor College of Medicine, Houston, TX USA; 4 Present Address: Proteovant Therapeutics, 2500 Renaissance Blvd, King of Prussia, PA USA

**Keywords:** Omics, Multi-omics, Visualization, Open-source, R, Bioinformatics, Genomics, Proteomics, Transcriptomics, Metabolomics

## Abstract

**Background:**

The results of high-throughput biology (‘omic’) experiments provide insight into biological mechanisms but can be challenging to explore, archive and share. The scale of these challenges continues to grow as omic research volume expands and multiple analytical technologies, bioinformatic pipelines, and visualization preferences have emerged. Multiple software applications exist that support omic study exploration and/or archival. However, an opportunity remains for open-source software that can archive and present the results of omic analyses with broad accommodation of study-specific analytical approaches and visualizations with useful exploration features.

**Results:**

We present OmicNavigator, an R package for the archival, visualization and interactive exploration of omic studies. OmicNavigator enables bioinformaticians to create web applications that interactively display their custom visualizations and analysis results linked with app-derived analytical tools, graphics, and tables. Studies created with OmicNavigator can be viewed within an interactive R session or hosted on a server for shared access.

**Conclusions:**

OmicNavigator can be found at https://github.com/abbvie-external/OmicNavigator

**Supplementary Information:**

The online version of this article (10.1186/s12859-024-05743-4) contains supplementary material, which is available to authorized users.

## Background

Software for the archival and interactive visualization of analyzed high-throughput biology (‘omic’) studies aids biomedical research by facilitating knowledge discovery across disciplines and time. A common workflow towards this end consists of bioinformaticians analyzing ‘raw’ data from instrumentation (e.g., next generation sequencing instruments) through formal hypothesis testing (e.g., H_o_: ‘there is an equal amount of gene X expressed in tissues Y and Z’), followed by sharing their analysis and visualizations with biology specialists via archival and visualization software. The biologist can then use the software to interact with the study results to gain insight and form mechanistic hypotheses. If more than one study is available, the biologist can interrogate and formulate hypotheses with archived data, too. In this way, the software increases the productivity of interactions between the biologist and the bioinformatician relative to ad-hoc approaches (email, spreadsheets, one-off presentations, etc.). Additionally, even in the absence of downstream non-computational stakeholders (e.g., the case where the analyst is also the biologist), computational analysts realize value from software that streamlines and reduces the burden of creating quality visualizations and data archives.

Multiple commercial and open-source software platforms exist that provide some combination of omic analysis, archival, and visualization functionality [1–5]. While such platforms may offer intuitive graphical user interfaces (GUIs) and advanced visualization and analysis functionality, they can be difficult to master and may lack the flexibility and functionality of scripting languages that contain popular bioinformatics packages such as R [6] and Python [7]. Given this, a common approach to analysis, visualization, and single-study archival that leverages R or Python directly is to capture a bioinformatic analysis and the results as an R Markdown [8] report or Jupyter notebook [9]. These documents are typically rendered as HTML files and usually contain embedded code, graphics, and tables. While these documents help make the analysis choices and code clear, using them for study result exploration and archival has drawbacks. First, the scripts used to construct a notebook (excluding the functions from the base scripting software and packages) may not be formalized into functions, documented, or versioned for reapplication in future analyses. Second, each notebook must be archived manually. Third, constructing advanced result exploration views that include interactive graphics and linked tables (dashboard functionality) requires skill with dedicated libraries (e.g. [10, 11]) or alternative programming languages like JavaScript. To facilitate the repeatable creation of these dashboards, pre-defined interactive graphics can be produced for the end-user following common bioinformatic workflows [12–14]. This model has been extended with web application hosting services that can accept uploaded data and guide the end-user through omic visualization and even analysis [2, 13, 15–17]. Despite the many options available, there are also drawbacks across these platforms. These include a focus on specific types of omic data, a lack of archival functionality, restrictions on analysis approach and/or plotting options, poor or little documentation, requirements to employ niche programming languages, and requirements to alter source code or databases for use (Additional file 1: Table S1).

Herein we present OmicNavigator, an R package for the archival and visualization of omic analyses. The software enables omic data analysts to create customizable web applications from the results of their work using only the statistical programming language, R. The OmicNavigator R package contains web application code, R functions for data deposition and retrieval, and a dedicated study container for the storage of measurements (e.g. RNAseq read counts), statistical analyses (differential expression and/or enrichment analysis), metadata, and custom plotting functions. Studies created with OmicNavigator are themselves R packages, accessible via a JavaScript web application that can be immediately interrogated on the user’s computer or deployed online to be explored by collaborators. The web application combines user-defined study results and feature plots with app-derived performant tables and interactive graphics common to bioinformatic analyses such as scatter, network, volcano, and barcode plots. The software also includes dynamic, multi-set filtering across hypothesis tests based on user-defined thresholds such as statistical significance or effect size.

## Implementation

All omic studies employ at least one mathematical model containing parameters that are estimated from high-throughput biology data. Hypothesis tests (e.g. of feature(s) equivalence across treatments, time, cellular differentiation states, etc.) using statistical inference approaches on these model parameters usually follow [18]. This natural ‘study-model-test’ hierarchy implies a universal data model applicable to all omic analyses and it is therefore employed within OmicNavigator (Additional file 2: Figs. S1 and S2). This framework enables nesting of multiple models within a study and multiple tests within a model, irrespective of their nature. The ‘study-model-test’ data model is therefore compatible with tests on individual or groups of biomolecules (typically referred to as ‘differential’ and ‘enrichment’ analysis, respectively), and it works equally well with ‘multi-omic’ studies where different biomolecule measurements (e.g., RNA, protein, metabolite) were modeled together or separately.

The OmicNavigator data model mirrors the analytical approach, wherein the ‘model’ object serves to group other data objects (Additional file 2: Fig. S2). For example, an analyst may model the same omic data (e.g., spectral counts from mass spectrometry proteomics data derived from three drug treatments of the same cell line) once with and once without a processing batch covariate. Such a study would contain two models of the same data and (assuming differences between each treatment are of interest) three tests within each model. In the common case of a ‘multi-omic’ study where the measurements are modeled separately, each omic dataset is naturally archived under a separate model.

**Fig. 1:**
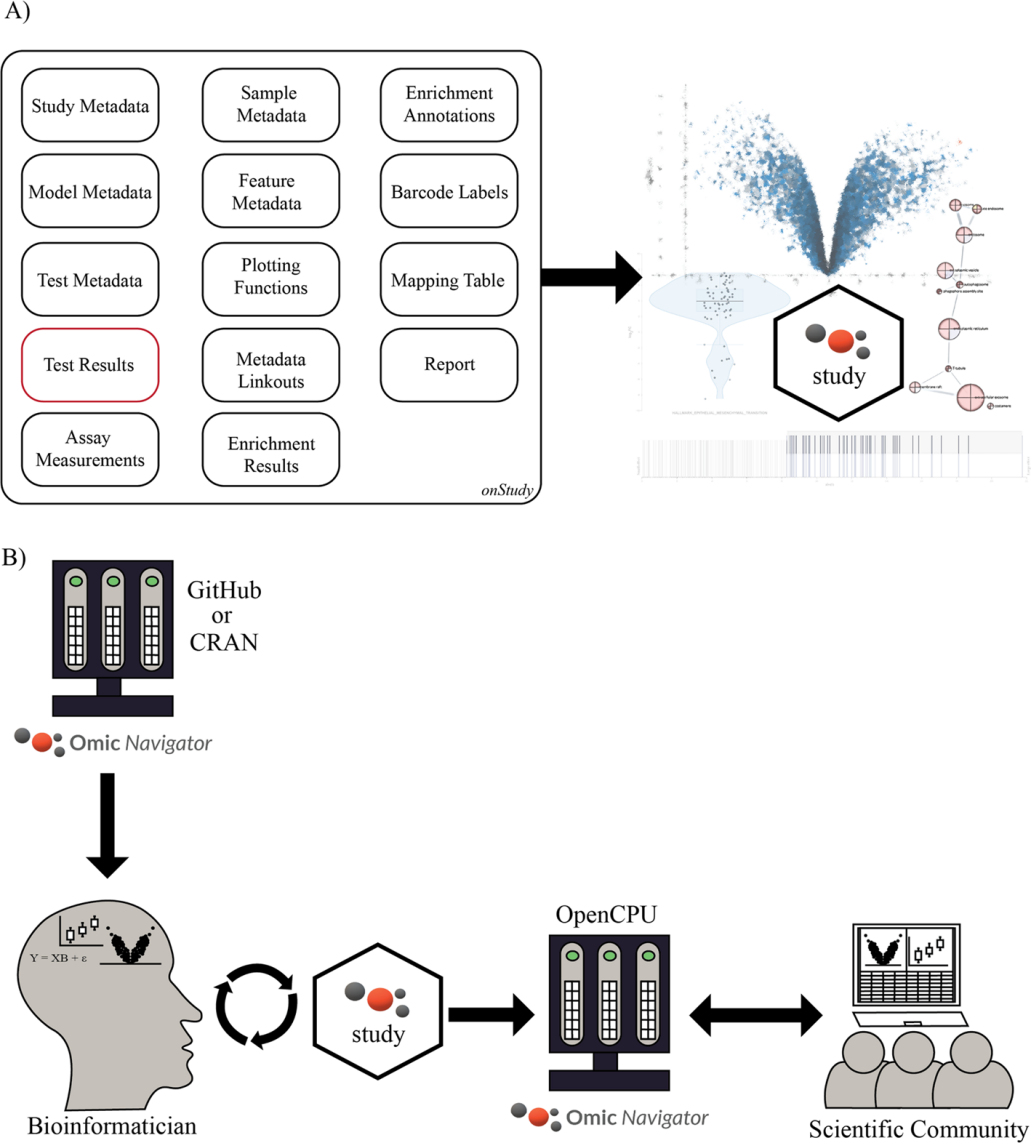
OmicNavigator study creation and hosting. **A** Data categories accepted by OmicNavigator software to produce a web application. OmicNavigator accepts unlimited study, model, and test metadata while requiring only Test Results (encircled in red) with a study, model, and test name. **B** Overview of OmicNavigator software usage. The bioinformatician may download the OmicNavigator R package from CRAN or GitHub to create a study package. The OmicNavigator study can be interrogated directly within a single-user server session or installed on a server for broader intra or internet access

The ‘study-model-test’ data model is encapsulated by the *onStudy* object within the OmicNavigator R package (Fig. 1A). The *onStudy* object accepts omic measurements (e.g., transcript counts), test results, feature (typically biomolecule) metadata, a mapping table between feature identifiers used in different models (useful for multi-omic studies), pathway/ontology data, links to external resources, a reproducible research report, and custom single and multi-feature plots. OmicNavigator comes with documented functions for data entry that may be used without regard to the model, statistical algorithm, or feature annotations (Ensembl, Uniprot, etc.) used for the analysis. Furthermore, OmicNavigator can display user-defined feature plots that bioinformaticians create with any plotting package dependency (e.g. [19]), including interactive plots constructed with the plotly package [10]. While OmicNavigator software works best with all *onStudy* objects populated, the minimal omic data required for an app is one test result *data frame* (Fig. 1A, red boxes).

When the *onStudy* object is populated, the end-user may call the *installStudy* function to convert the *onStudy* object into an R package and install it in the specified library path (note there is also an *exportStudy* function for packaging results into an R package tarball for easy sharing with collaborators). Following installation, the study results are immediately accessible for viewing within the OmicNavigator app. The end-user can access the app via a single-user server within an interactive R session by calling the *startApp* function or online via a multi-user OpenCPU server [20]. The latter setup is recommended for laboratories or organizations hosting multiple omic studies. The process of creating and hosting OmicNavigator studies is depicted in Fig. 1B.

Below, we demonstrate most OmicNavigator app features with a public RNA-seq dataset derived from three mouse mammary cell populations (basal, luminal progenitor (LP), and mature luminal (ML)) measured in triplicate [21]. We analyzed this data with the algorithms limma-voom and camera [22, 23], adapting the code from the Bioconductor workflow entitled ‘RNAseq123’ where possible [24]. Data, Analysis, and OmicNavigator build scripts for this study can be found at https://github.com/abbvie-external/OmicNavigatorExample. Furthermore, to demonstrate multi-omic study support, we created a second study using protein abundance and phosphorylation measurements captured following SARS-Cov-2 infection of Vero E6 cells [25]. Data and OmicNavigator build scripts for this study can be found at https://github.com/abbvie-external/OmicNavigatorMultimodelExample.

OmicNavigator displays the results of an analysis in two tabs, ‘differential’ for single-feature results and ‘enrichment’ for test results on groups of features. Within the ‘differential’ tab (Additional file 2: Fig. S3), the study ‘RNAseq123’ has one model called ‘Differential_Expression’, wherein three tests (‘BasalvsLP’, ‘BasalvsML’, and ‘LPvsML’) of pairwise transcript differential expression across the cell populations were applied. Upon selection of a ‘study-model-test’ combination, the differential tab displays the data provided by the end-user as a linked scatter, table, and feature viewer. Single or multi-feature selections are highlighted in the scatter plot and table while user-created plots are rendered in the single or multi-feature plotting windows. All images produced by the app are exportable as PNG or SVG files and all table views are exportable as txt or xls files. The ‘differential’ tab features a performant table with single and multi-feature search and extensive column filtering capabilities. The table also includes dynamically rendered favicons linking to feature-level external resources [26]. The exportable scatterplot employs hex binning to enable the plotting of large omic datasets and allows for zooming, selecting, and labeling features. The scatterplot also dynamically alters feature opacity and functionality in response to table or ‘set analysis’ (see below) filter operations. In this way, the app provides advanced table and scatterplot functionality that is interactively linked with each other and with custom feature plots specific to the study-model-test selected. The app functionality within the differential tab is captured in movies Additional files 3,4,5,6.

Biologically interesting features are often identified within intersections, unions, and complements of sets of omic hypothesis test results. For example, it is often of interest to identify genes that are differentially expressed in disease cell type A relative to a control cell type (set 1) but not differentially expressed in disease cell type A relative to disease cell type B (set 2). OmicNavigator enables the discovery of these interesting sets of features via the ‘Set Analysis’ toggle (Additional file 2: Fig. S4). Users may specify set definitions based on e.g., p-value and effect size before applying a filter. Features that pass the multi-set filtering operation remain within the interactive table and scatter plot. Notably, the app also returns an overview ‘UpSet’ plot displaying the top intersecting sets of features across all hypothesis tests applied within the selected model using the R package ‘upsetR’ [27, 28]. Set filtering is demonstrated in the movie Additional file 7.

Tests performed on features grouped by a common theme such as pathway membership, function, or location are also commonly performed in omic analyses [23, 29–31]. These ‘enrichment’ analyses are undertaken to identify biological functions differentially regulated by the coordinated activity changes of many (often multi-functional) features. OmicNavigator supports the archival and visualization of the results of these group-tests within the ‘Enrichment’ tab of the app (Additional file 2: Fig. S5). Here, the end-user selects the ‘model’ specifying the tests applied (these tests must match or be a subset of those applied in the single feature case) and ‘database’ (databases commonly employed for this purpose include the Gene Ontology [32] and Reactome [33]) specifying the grouping of features into ‘terms’. Upon selection, a table is provided to the end-user with p-values for each term-test combination. This table is also amenable to ‘Set Analysis’.

While useful for an overview of differentially regulated terms, a table provides no information about (1) the relative variability of individual features within a term and (2) how many features are shared between terms. This information is critical for translating the results of omic enrichment analyses into intuition about biological processes affected by experimental conditions and designing follow-up molecular biology experiments that target single features. OmicNavigator addresses the first issue with interactive barcode plots and the second with interactive network plots. ‘Barcode’ plots are a commonly applied visualization of enrichment analysis results [30, 34] and are meant to convey a global view of multiple features within a term, ranked according to some test statistic (here the absolute value of the t-statistic). Network plots convey similarity of terms by depicting them as nodes, potentially connected into groups by edges with weights defined by a similarity measure.

Within OmicNavigator, a barcode plot is produced upon clicking a p-value within the enrichment results table. The interactive barcode, box and feature plot is produced using test result information from each feature within the selected term-test combination (Additional file 2: Fig. S6). In this way, the biologist end-user can dynamically view all features within the term, appreciating outliers or biological themes inferred from knowledge of feature (typically gene) function. A network view modeled on the Cytoscape plugin EnrichmentMap is also available [35, 36]. Here, each node is a term colored by test result p-value and sized by feature count (Additional file 2: Fig. S7). If multiple tests were applied, each is represented as a colored wedge within the node. Similarity across terms is captured as edges defined by Jaccard and/or Overlap coefficients. The network layout dynamically and flexibly sorts and arranges nodes/node clusters in tiles, ensuring that the largest clusters (defined by node or edge count) or clusters containing the most significantly regulated terms are prominently displayed. The tiled nature of the network layout naturally creates adequate spacing between nodes or node-clusters that vary in count and/or node size. Additionally, the layout can be sorted to reveal a general trend when viewed from top to bottom then left to right. By clicking on a node or wedge, the end-user can access the barcode plot view for that term.

The app functionality within the enrichment tab is captured in the movies Additional files 8 and 9.

## Discussion

Within our company, OmicNavigator is used to convey the results of bioinformatic analyses and facilitate their interactive exploration. Our internal server hosts hundreds of OmicNavigator studies and is frequently accessed. Often-cited achievements resulting from the use of OmicNavigator can be found in Additional file 10.

We designed OmicNavigator to be intuitive and easy to use for bioinformaticians and biologists. OmicNavigator presents a consistent dashboard for the visualization of diverse omic studies. This consistent presentation may simplify and reduce the training needed for biologists when they return to the app for each new study while maintaining the visualization and modeling choices of the bioinformatician. Although already built to be intuitive, the scatterplot and table have ‘info’ buttons to provide guidance while advanced features like set analysis and network views are hidden by toggles, allowing the biologist to ignore that functionality if desired. Each figure and field within the table may have a tool tip description produced by the analyst. Moreover, the ‘analysis details’ button enables easy review of e.g., the study design and analysis choices for additional context. Only two OmicNavigator function calls (*createStudy* and *addResults*) are required to create a study, and the bioinformatician may add or subtract to a modular OmicNavigator study at their leisure. Over time, OmicNavigator functions can be naturally added to an R analysis pipeline to streamline iterative study creation.

One of the challenges we faced developing OmicNavigator was to create an architecture capable of handling a high volume of heterogeneous data while enabling maximum interactivity between application components. To that end, we chose the JavaScript libraries React [37] and D3 [38]. React helped to overcome the challenge of data management through declarative views and state management, and D3 enabled the development of complex visual components. Both libraries were instrumental in addressing the challenge of interactivity.

A server hosting OmicNavigator can support thousands of omic studies for online access and integration with other applications. Importantly, OmicNavigator was built to comply with findability, accessibility, interoperability, and reproducibility (FAIR) principles [39]. First, OmicNavigator studies are R packages that may contain metadata to support findability. Second, OmicNavigator has an extensive set of data accessor functions which can be used over HTTP with OpenCPU. The OpenCPUserver hosts OmicNavigator and exposes an API to R with powerful data transformation capabilities [20, 40]. Thus, tabular data can be returned in multiple formats (e.g. txt, csv, JSON, feather) and plots can be returned using R graphics file devices in any desired format (e.g. pdf, svg, jpeg, tiff) and size, supporting accessibility and interoperability. Last, OmicNavigator studies may include a report outlining the analysis undertaken, thereby supporting reproducibility. Taken together, these attributes enable FAIR OmicNavigator deployment where study data and plots are readily accessible from the web application and the OpenCPU HTTP API.

OmicNavigator may expand upon or refine existing features in future releases. While currently unconstrained, metadata about studies, models, tests, and assays can be standardized and paired with controlled vocabularies to facilitate study finding and meta-analyses. The creation of metadata capture and study builder features would improve data quality and facilitate construction of OmicNavigator studies by analysts with less coding experience, respectively. Within a study, multi-omic (or multi-model) functionality can be improved by expanding set analysis functionality to enable filtering features across a set of models/datasets. Enrichment analysis functionality can also be improved with the addition of custom multi-feature plots and the support of pathway diagrams.

## Conclusions

OmicNavigator is an R package for omic study storage and visualization that can be used to create custom, study-specific interactive web applications using only R. OmicNavigator studies can be single or multi-omic, and the web application comes with advanced visualization features such as set analysis, network views, and dynamic barcode and scatter plots. Studies created with OmicNavigator are also R packages that can be easily shared with collaborators or installed on a server running OpenCPU for intra/internet access.

## Availability and requirements

Project name: OmicNavigator, Project home page: https://github.com/abbvie-external/OmicNavigator, Operating system(s): Platform independent.

Programming language: R, Other requirements: R >  = 3.2.0, License: MIT.

Any restrictions to use by non-academics: none.

### Supplementary Information

**Additional file 1**. Table comparing select omic archival, exploration, and/or visualization software.

**Additional file 2**. Figures depicting data model and app functionality.

**Additional file 3**. Overview of differential analysis tab.

**Additional file 4**. Movie of interactive scatterplot functionality.

**Additional file 5**. Movie of interactive table functionality.

**Additional file 6**. Movie of custom plotting functionality.

**Additional file 7**. Movie of set analysis functionality.

**Additional file 8**. Overview of enrichment analysis functionality, table, and barcode plot.

**Additional file 9**. Movie of enrichment analysis network functionality.

**Additional file 10**. Bulleted list of achievements (success stories) derived from using OmicNavigator at AbbVie.

## Data Availability

The OmicNavigator software is available from the GitHub repository, https://github.com/abbvie-external/omicnavigator. The creation data and scripts for the ‘RNAseq123’ OmicNavigator demo study is available from the GitHub repository, https://github.com/abbvie-external/OmicNavigatorExample. The creation data and scripts for the ‘SARSCoV2.proteomic.and.phosphoproteomic.profiling’ demo study is available from the GitHub repository, https://github.com/abbvie-external/OmicNavigatorMultimodelExample.

## Abbreviations

Omic: Term for high-throughput biomolecule measurement technologies
GUI: Graphical user interface
HTTP: Hypertext transfer protocol
LP: Luminal progenitor
ML: Mature luminal
FAIR: Findable, accessible, interoperable, reproducible
JSON: JavaScript object notation
RNA: Ribonucleic acid
API: Application programming interface
pdf: Portable document format
txt: Text document
xls: Microsoft excel worksheet
