# Supplementary material for: OmicNavigator: open-source software for the exploration, visualization, and archival of omic studies
Source: BMC Bioinformatics. 2024 Apr 24;25:162. doi: 10.1186/s12859-024-05743-4 (PMC11040775; doi:10.1186/s12859-024-05743-4)
Supplement: Supplementary file 2 — Additional file 2. Figures depicting data model and app functionality. [file 12859_2024_5743_MOESM2_ESM.pdf]

Figure S1

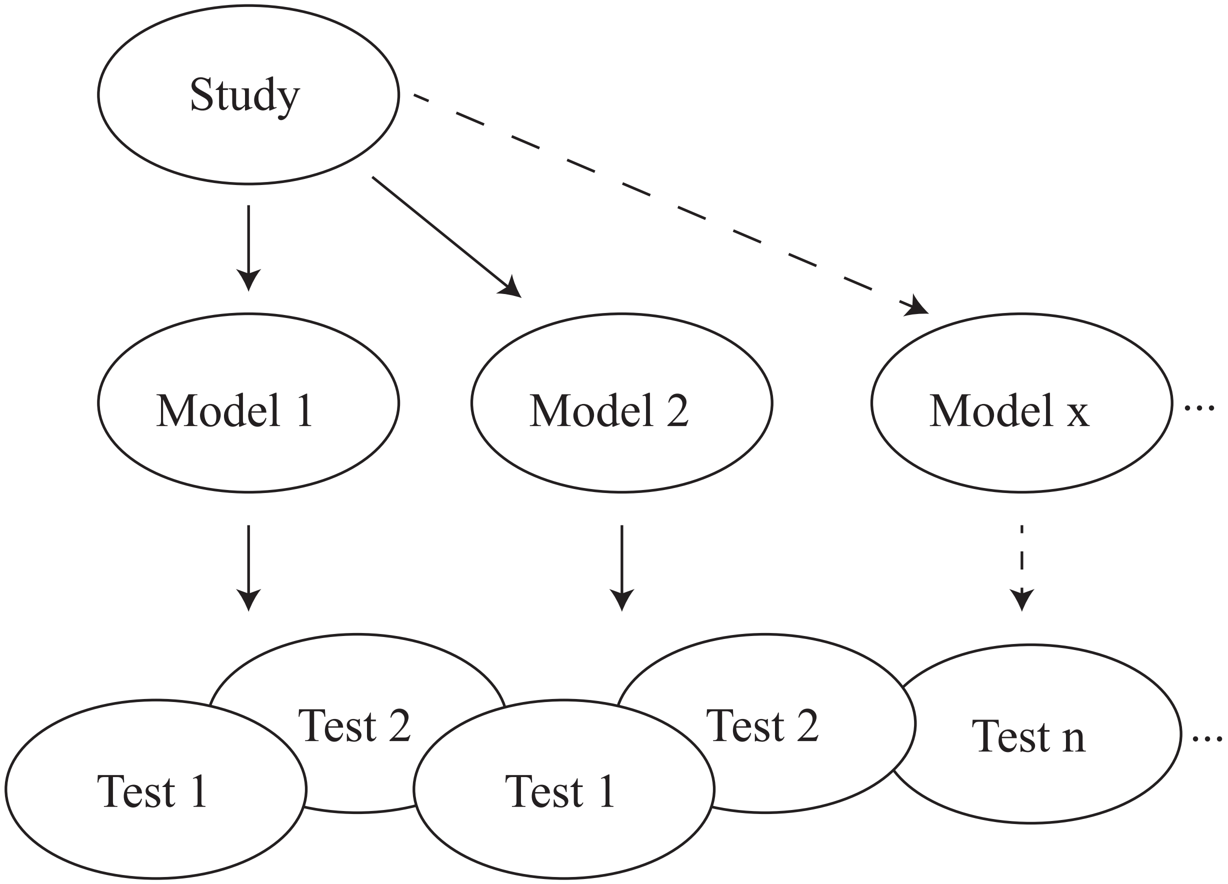

Figure S2

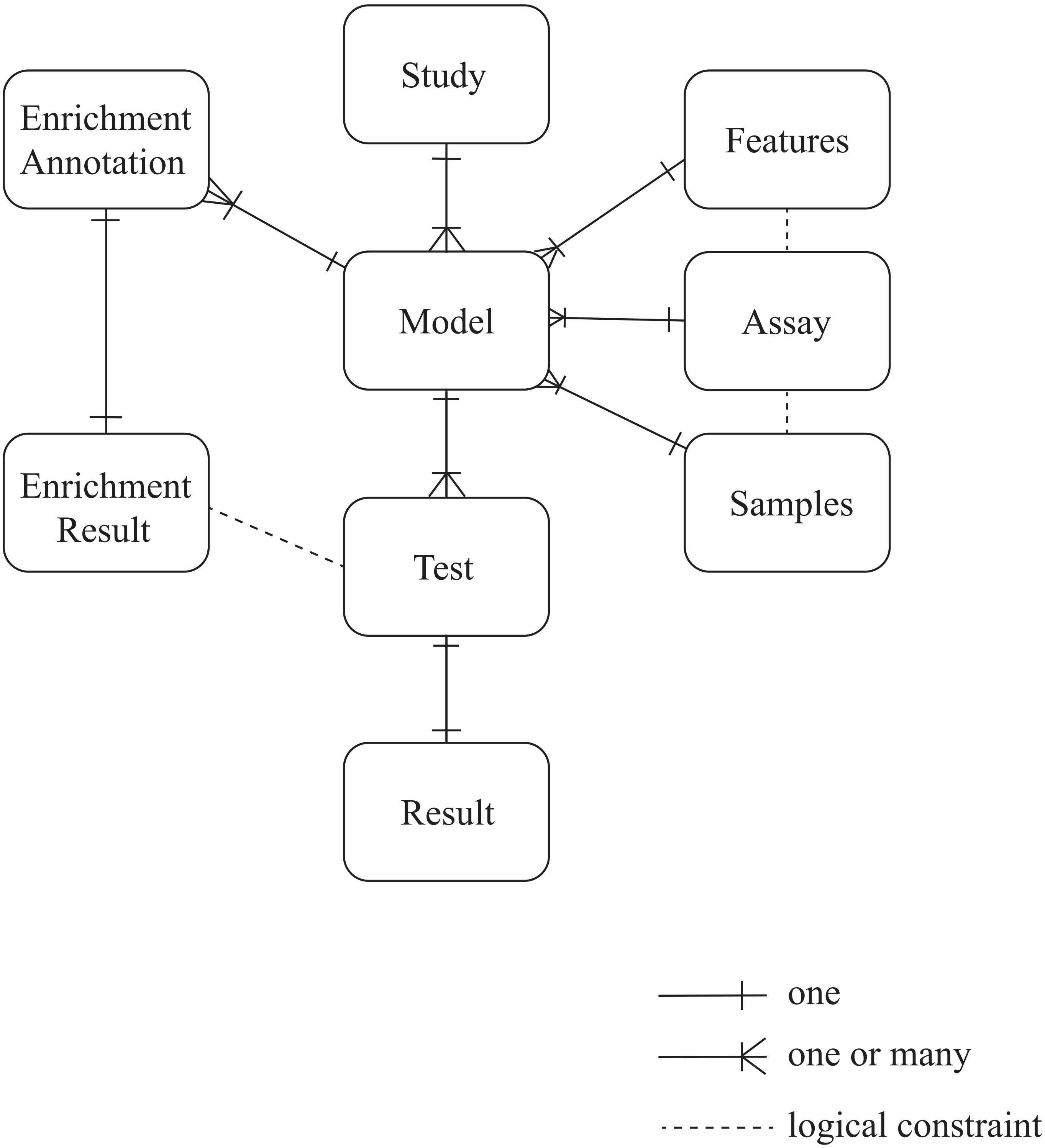

Study
Enrichment Annotation
Features
Model
Assay
Enrichment Result
Samples
Test
Result
one
one or many
logical constraint

Figure S3

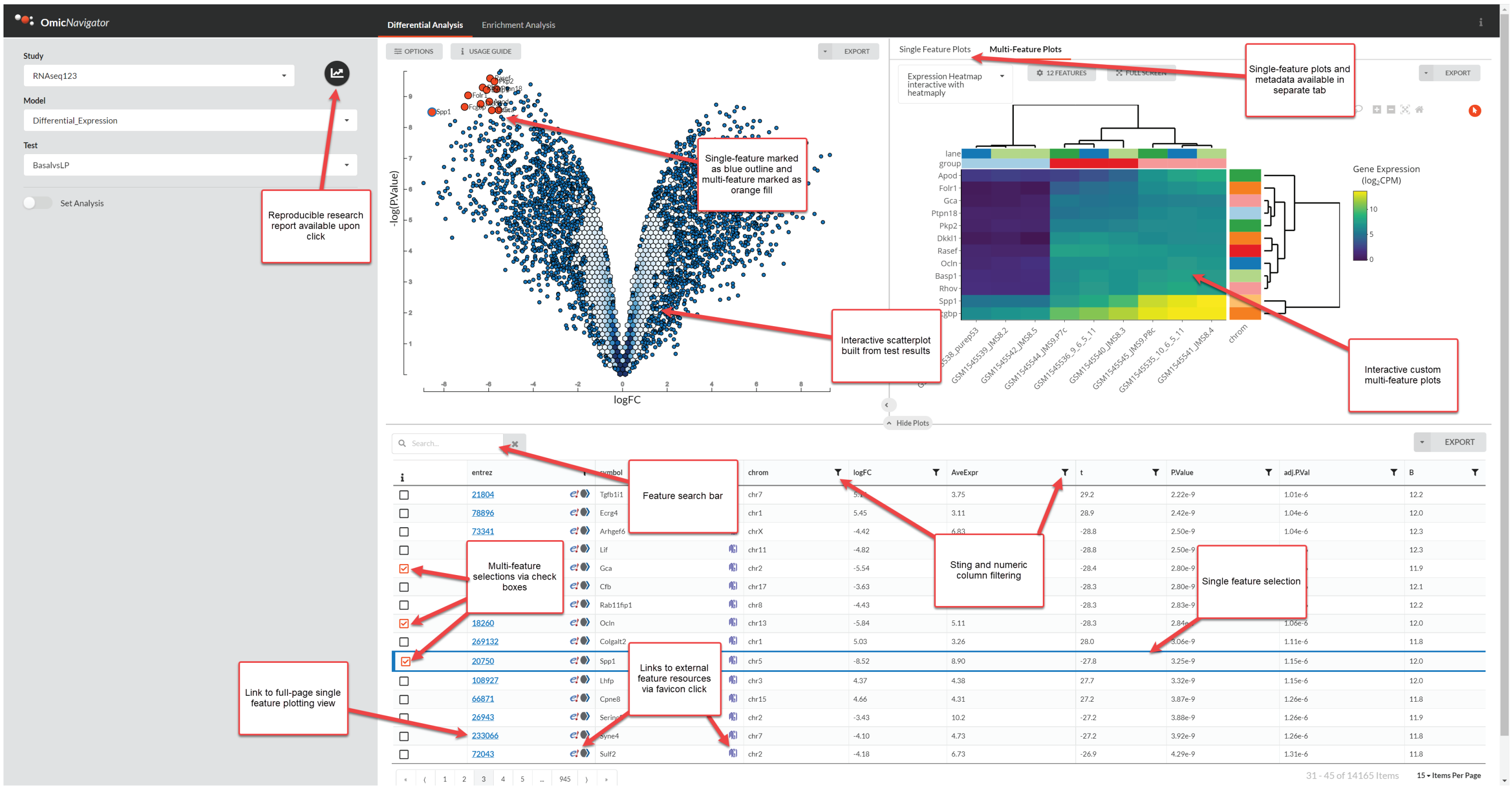
OmicNavigator
Differential Analysis
Enrichment Analysis
Study
RNAseq123
Model
Differential_Expression
Test
BasalvsLP
Set Analysis
Reproducible research report available upon click
Single-feature marked as blue outline and multi-feature marked as orange fill
Single Feature Plots
Multi-Feature Plots
Single-feature plots and metadata available in separate tab
Expression Heatmap interactive with heatmaply
Gene Expression
logFC
-log(PValue)
Interactive scatterplot built from test results
Interactive custom multi-feature plots
Hide Plots
EXPORT
Feature search bar
Multi-feature selections via check boxes
Sting and numeric column filtering
Single feature selection
Links to external feature resources via favicon click
Link to full-page single feature plotting view
31 - 45 of 14165 Items
15 Items Per Page

Figure S4

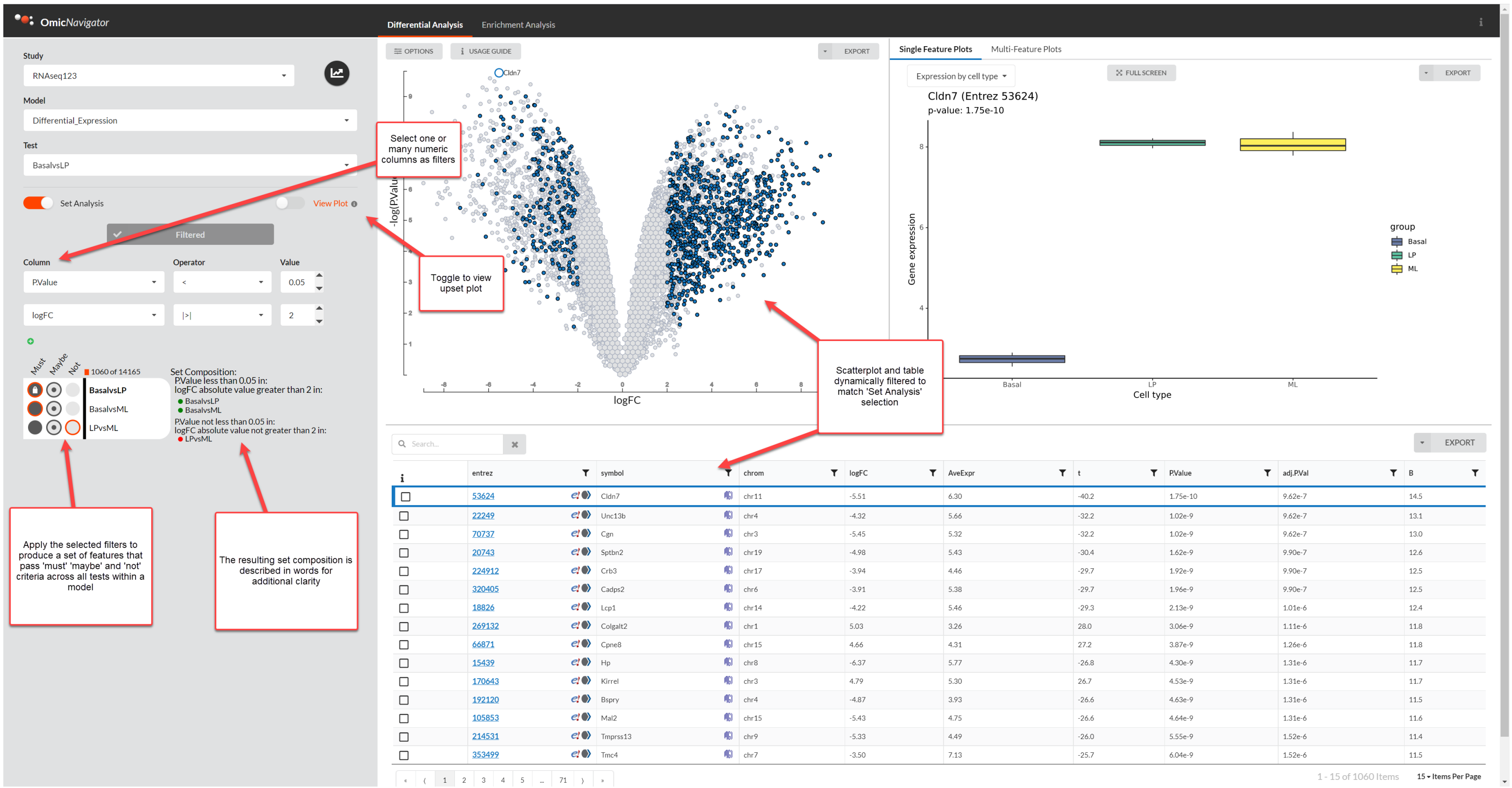

OmicNavigator
Differential Analysis
Enrichment Analysis
Study
RNAseq123
Model
Differential_Expression
Test
BasalvsLP
Set Analysis
View Plot
Filtered
Column
Operator
Value
PValue
<
0.05
logFC
|>|
2
Must
Maybe
Not
1060 of 14165
BasalvsLP
BasalvsML
LPvsML
Set Composition:
PValue less than 0.05 in:
logFC absolute value greater than 2 in:
BasalvsLP
BasalvsML
PValue not less than 0.05 in:
logFC absolute value not greater than 2 in:
LPvsML
Select one or many numeric columns as filters
Toggle to view upset plot
Scatterplot and table dynamically filtered to match 'Set Analysis' selection
Apply the selected filters to produce a set of features that pass 'must' 'maybe' and 'not' criteria across all tests within a model
The resulting set composition is described in words for additional clarity
OPTIONS
USAGE GUIDE
EXPORT
Cldn7
logFC
Single Feature Plots
Multi-Feature Plots
Expression by cell type
FULL SCREEN
Cldn7 (Entrez 53624)
p-value: 1.75e-10
Gene expression
group
Basal
LP
ML
Cell type
entrez
symbol
chrom
logFC
AveExpr
t
PValue
adj.PVal
B
1 - 15 of 1060 Items
15 Items Per Page

Figure S5

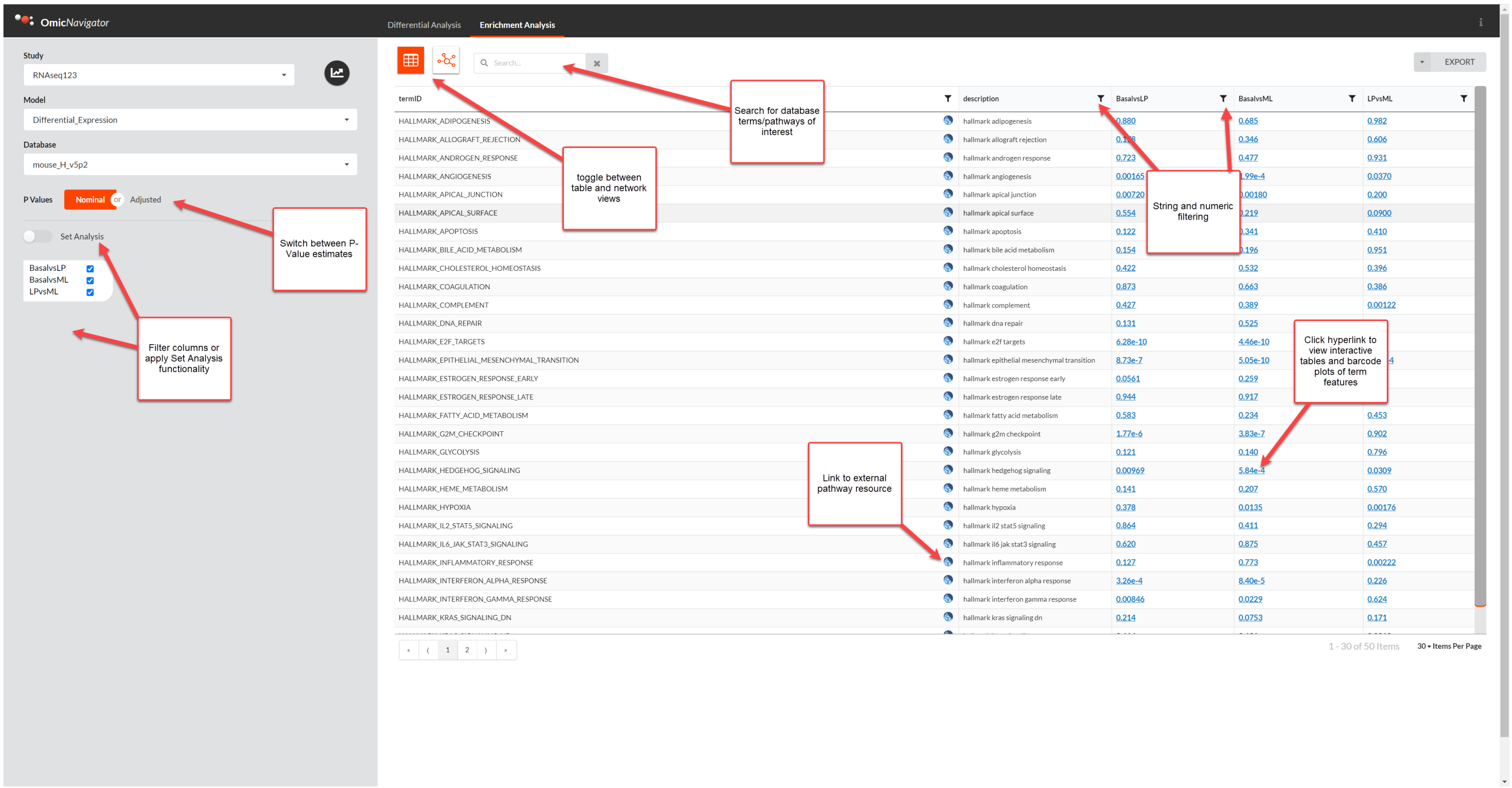
OmicNavigator
Differential Analysis
Enrichment Analysis
Study
RNAseq123
Model
Differential_Expression
Database
mouse_H_v5p2
P Values
Nominal
Adjusted
Set Analysis
BasalvsLP
BasalvsML
LPvsML
EXPORT
termID
description
BasalvsLP
BasalvsML
LPvsML
Search for database terms/pathways of interest
toggle between table and network views
Switch between P-Value estimates
String and numeric filtering
Filter columns or apply Set Analysis functionality
Click hyperlink to view interactive tables and barcode plots of term features
Link to external pathway resource
1 - 30 of 50 Items
30 Items Per Page

Figure S6

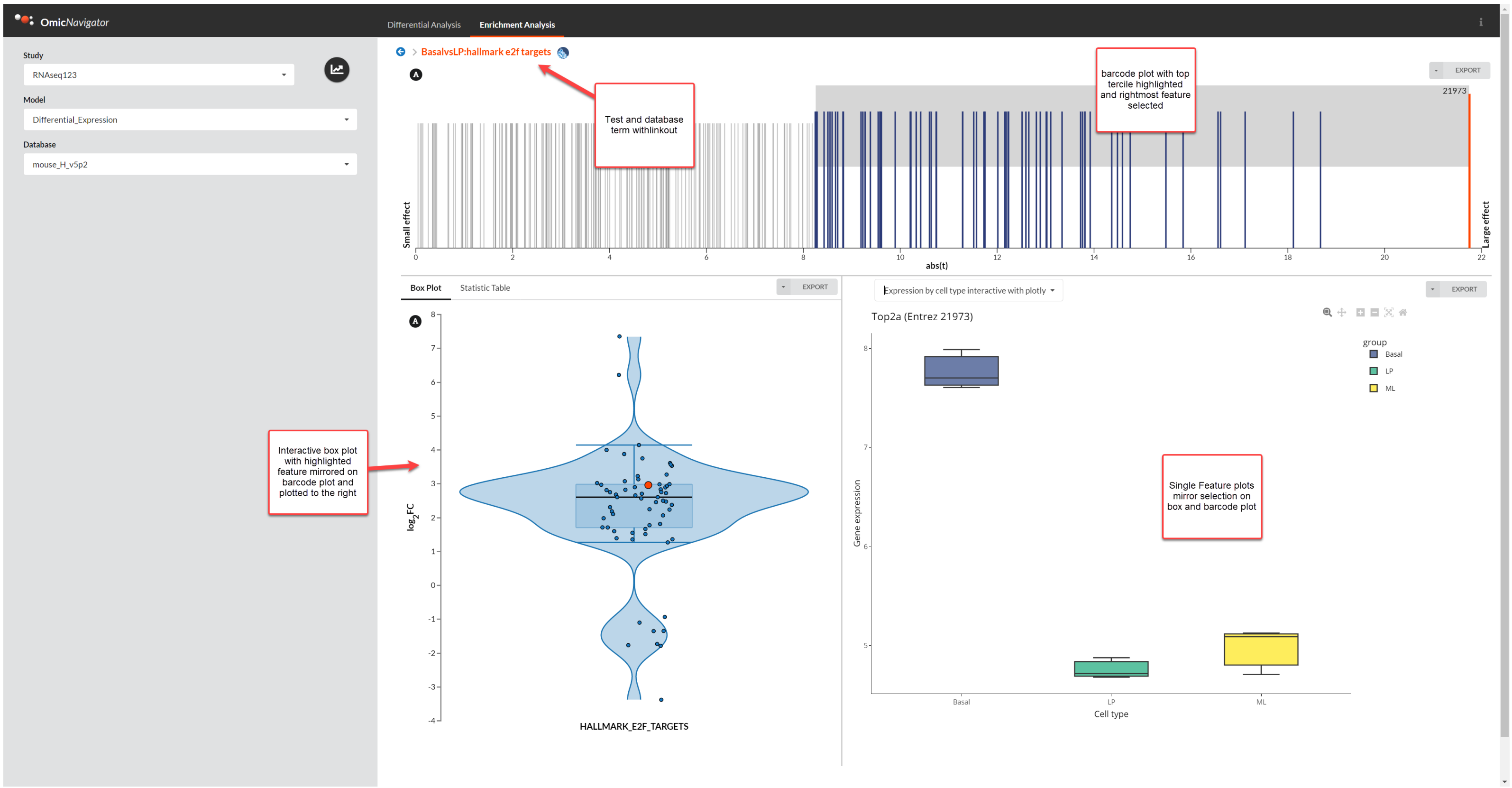
OmicNavigator
Differential Analysis
Enrichment Analysis
Study
RNAseq123
Model
Differential_Expression
Database
mouse_H_v5p2
BasalvsLP:hallmark e2f targets
Test and database term withlinkout
barcode plot with top tercile highlighted and rightmost feature selected
EXPORT
21973
Small effect
Large effect
abs(t)
Box Plot
Statistic Table
EXPORT
Expression by cell type interactive with plotly
EXPORT
Top2a (Entrez 21973)
group
Basal
LP
ML
Interactive box plot with highlighted feature mirrored on barcode plot and plotted to the right
log2FC
HALLMARK_E2F_TARGETS
Single Feature plots mirror selection on box and barcode plot
Gene expression
Basal
LP
ML
Cell type

Figure S7

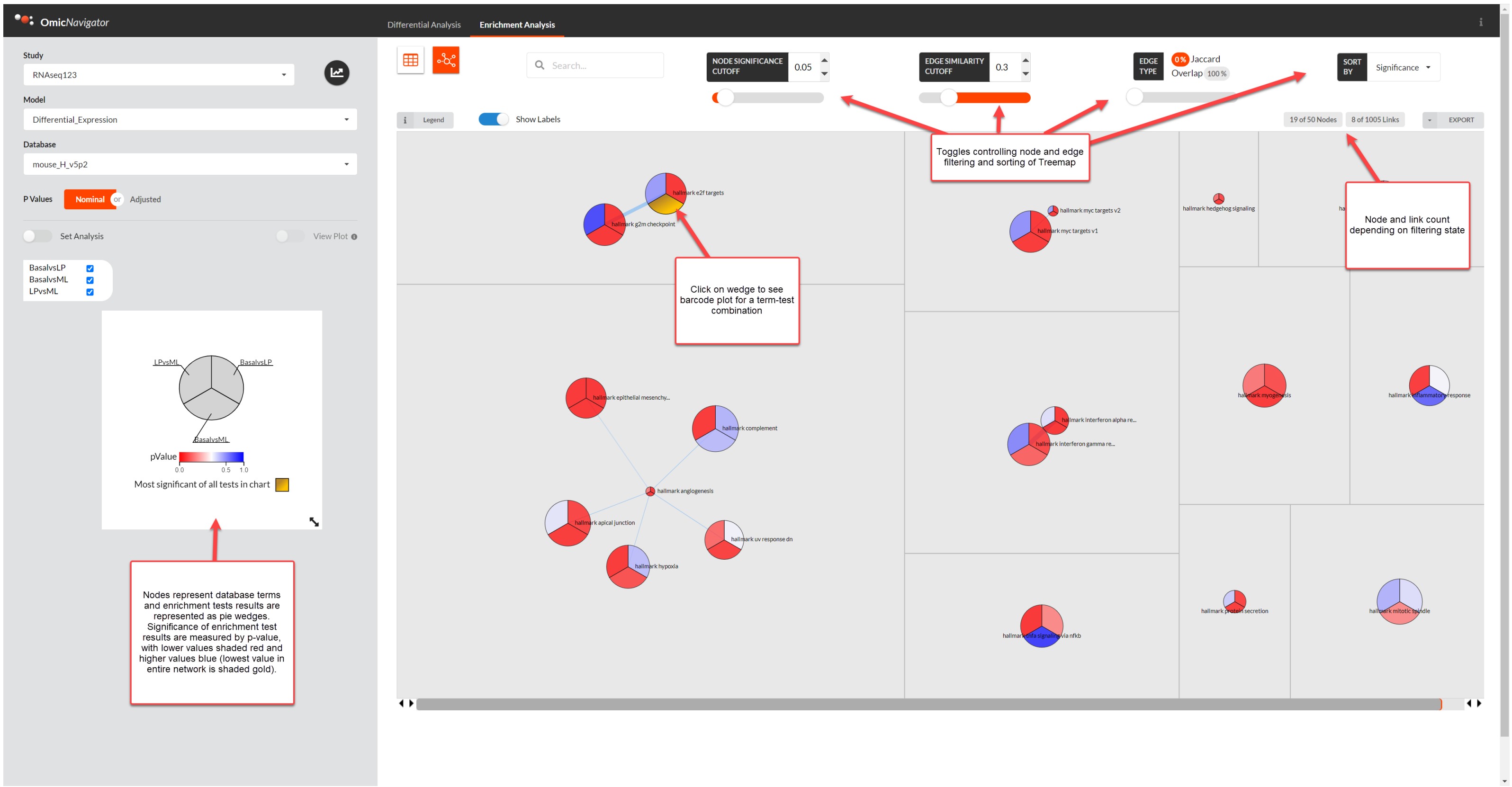

OmicNavigator
Differential Analysis
Enrichment Analysis
Study
RNAseq123
Model
Differential_Expression
Database
mouse_H_v5p2
P Values
Nominal
Adjusted
Set Analysis
View Plot
BasalvsLP
BasalvsML
LPvsML
pValue
Most significant of all tests in chart
NODE SIGNIFICANCE CUTOFF
0.05
EDGE SIMILARITY CUTOFF
0.3
EDGE TYPE
Jaccard
Overlap
SORT BY
Significance
Legend
Show Labels
19 of 50 Nodes
8 of 1005 Links
EXPORT
Toggles controlling node and edge filtering and sorting of Treemap
Click on wedge to see barcode plot for a term-test combination
Node and link count depending on filtering state
Nodes represent database terms and enrichment tests results are represented as pie wedges. Significance of enrichment test results are measured by p-value, with lower values shaded red and higher values blue (lowest value in entire network is shaded gold).

Figure S1 OmicNavigator Framework. An OmicNavigator study may contain arbitrarily many models. There can be arbitrarily many tests within each model.

Figure S2 OmicNavigator Conceptual Data Model. Primary OmicNavigator objects and relationships. Note that 'Enrichment Annotations' are referred to as 'Databases' within the app. The row and column names of the 'Assays' object must match the featureIDs within the 'Features' object and sampleIDs within the 'Samples' object, respectively. The 'Enrichment Results' object must be grouped by testID as captured within the 'Test' object.

Figure S3 OmicNavigator Differential Analysis Tab. The state of the app following selection of multiple genes is displayed with informational text boxes explaining app functionality.

Figure S4 OmicNavigator Set Analysis. The state of the app following application of the 'set analysis' feature is shown with informational text boxes explaining app functionality.

Figure S5 OmicNavigator Enrichment Analysis Tab. The state of the app following selection of a 'database', or enrichment annotation is shown with informational text boxes explaining app functionality.

Figure S6 OmicNavigator Enrichment Analysis Barcode View. The state of the app following selection of a term within a 'database', or enrichment annotation with informational text boxes explaining app functionality.

Figure S7 OmicNavigator Enrichment Analysis Network View. The state of the app following selection of a 'database', or enrichment annotation and selecting the network view button is shown with informational text boxes explaining app functionality.
