# Supplementary material for: OmicNavigator: open-source software for the exploration, visualization, and archival of omic studies
Source: BMC Bioinformatics. 2024 Apr 24;25:162. doi: 10.1186/s12859-024-05743-4 (PMC11040775; doi:10.1186/s12859-024-05743-4)
Supplement: Supplementary file 10 — Additional file 10. Bulleted list of achievements (success stories) derived from using OmicNavigator at AbbVie. [file 12859_2024_5743_MOESM10_ESM.docx]

Often-cited achievements resulting from OmicNavigator use include the following:

- OmicNavigator enables biologist end-users to explore results independently from the analyst (typically following an initial presentation from the analyst to the biologists partnering on the study) to formulate mechanistic insights and hypotheses. This achieves significant time efficiency gains for analysts and biologists.
  - “Collaborators from different fields seem to find the software intuitive since after learning Omic Navigator’s functionality they do not reach back out with follow up questions, but instead will share back Omic Navigator plots they have generated themselves.” – an OmicNavigator end-user.
  - “Previously, I used to create all the plots for our collaborators, but now OmicNavigator handles everything.” – an OmicNavigator end-user.
  - “We’ve employed OmicNavigator to assist in validating the expression of over 300 candidate target genes leading to the advancement of 16 of these for *in vitro* biological functional studies…” – an OmicNavigator end-user.
- The consistent interface and functionality and data presentation lessens biologist end-user learning curve for new studies or dependency upon a particular analyst’s presentation style.
  - “Omic Navigator has greatly improved the efficiency of collaboration with additional biological scientists, as they are already familiar with working with the software and do not need to adapt to my personal style of presenting results. By standardizing visualization methods and data archiving, we have overcome previous challenges in terms of user-friendly data retrieval.” – an OmicNavigator end-user
- The ability to rapidly identify features of interest and export plots and tables for downstream use. Previously, this required explicit communication between the analyst and the biologist, thereby achieving time savings.
  - “Multiple collaborators have now exported Omic Navigator plots directly for incorporation into their manuscripts to be submitted for publication.” – an OmicNavigator end-user.
- Streamlined study creation with an accessible learning curve for those less fluent in R.
  - “With minimal training scientists in our lab have been able to learn the basic requirements for formatting data and utilizing the visualization capabilities Omic Navigator incorporates, even if they are not strongly experienced in R programming.” – an OmcNavigator end-user.
- Scriptable study creation and pipeline readiness.
  - “I particularly appreciate how seamlessly I can generate OmicNavigator packages programmatically once I have developed the build script. The example scripts provided on GitHub have been a valuable resource, as they offer a helpful starting point to understand the data structure and capabilities of Omic Navigator. Furthermore, these scripts are flexible enough to allow me to add custom plots that my collaborators are accustomed to seeing.” – an OmicNavigator end-user
- The ability to combine results from different omic measurements, models, or analysts into multi-omic and/or multi-model studies.
  - OmicNavigator helps to achieve efficient collaboration across different departments in this way because different omic measurements are often collected and analyzed by different groups. OmicNavigator has functions called ‘importStudy’ and ‘combineStudy’ that enable this achievement when each group creates an independent OmicNavigator study.
- Efficient and faithful omic data transfer internally and externally.
  - OmicNavigator leverages the R package system for study creation (an OmicNavigator study is an R package bundle) so they are easily shared.
    - “Creating an OmicNavigator study is straightforward, and each study is packaged into an R package, facilitating the sharing of study results. With this tool, bioinformaticians retain full control over the analysis process, while biologists can conveniently visualize the results and draw their own conclusions.” – an OmicNavigator end-user.
  - Sharing and editing of a study does not require access to source data and a build script for every team. The ‘importStudy’ function allows users to install the package and work from the study S3 object directly.
  - External collaborators have sent analysis, plots, and data in the form of OmicNavigator studies, thereby saving weeks of work to transform custom formats into an internal standard.
